# Молекулярно-генетические аномалии в кортикотропных опухолях гипофиза: фундаментальные исследования и перспективы использования в клинической практике

**DOI:** 10.14341/probl13273

**Published:** 2023-11-02

**Authors:** А. М. Лапшина

**Affiliations:** Национальный медицинский исследовательский центр эндокринологии

**Keywords:** АКТГ-секретирующая аденома гипофиза, молекулярные маркеры, экзом, транскриптом

## Abstract

За последние годы проводится большое количество исследований по изучению молекулярно-генетических аномалий в АКТГ-секретирующих опухолях гипофиза. В данном обзоре представлен комплексный анализ результатов исследований полного экзома (герминальные и соматические мутации, хромосомные аномалии в кортикотропиномах, развившихся в составе наследственных синдромов МЭН 1, 2, 4, DICER1, комплекс Карни и пр., и изолированных опухолях соответственно) и транскриптома (специфичные профили экспрессии генов гормонально активных и неактивных кортикотропином, регуляция клеточных циклов и сигнальных путей). Современные технологии (секвенирование следующего поколения — NGS) позволяют изучить состояние микроРНКома, ДНК метилома. Таким образом, показан широкий спектр фундаментальных исследований, результаты которых позволяют определить и осмыслить ключевые, ранее известные и новые механизмы патогенеза и биомаркеры кортикотропином. Дана характеристика наиболее перспективным молекулярно-генетическим факторам, которые возможно использовать в клинической практике для скрининга и более ранней диагностики наследственных синдромов и изолированных кортикотропином, дифференциальной диагностики различных форм эндогенного гиперкортицизма, чувствительности к уже существующим и потенциальным видам терапии и персонализированного определения исхода болезни Иценко-Кушинга.

## Abstract (en)

In recent years, a large number of studies have been carried out to research molecular genetic abnormalities in ACTH-­secreting pituitary tumors. This review presents a comprehensive analysis of exome studies results (germline and somatic mutations, chromosomal abnormalities in corticotropinomas which developed as part of hereditary syndromes MEN 1, 2, 4, DICER1, Carney complex etc., and isolated tumors, respectively) and transcriptome (specific genes expression profiles in hormonally active and inactive corticotropinomas, regulation of cell cycles and signal pathways). Modern technologies (next-generation sequencing — NGS) allow us to study the state of the microRNAome, DNA methylome and inactive chromatin sites, in particular using RNA sequencing. Thus, a wide range of fundamental studies is shown, the results of which allow us to identify and comprehend the key previously known and new pathogenesis mechanisms and biomarkers of corticotropinomas. The characteristics of the most promising molecular genetic factors that can be used in clinical practice for screening and earlier diagnosis of hereditary syndromes and isolated corticotropinomas, differential diagnosis of various forms of endogenous hypercorticism, sensitivity to existing and potential therapies and personalized outcome determination of Cushing`s disease.

## ВВЕДЕНИЕ

За последние годы был достигнут значительный прогресс в понимании молекулярно-биологических аномалий в кортикотропиномах и других опухолях гипофиза. К ним относят генетические и эпигенетические факторы, драйверные соматические мутации, вариабельность количества нуклеотидов в составе хромосом, метилирование генов, регуляции микроРНК и факторов транскрипции.

Болезнь Иценко-Кушига — это тяжелое многосимптомное заболевание гипоталамо-гипофизарного происхождения, вызванное АКТГ-секретирующей опухолью гипофиза (кортикотропиномой), которая стимулирует избыточную секрецию гормонов коры надпочечников. Среди всех опухолей гипофиза кортикотропиномы составляют 10–15%. Методом выбора лечения является нейрохирургическое удаление опухоли. Кортикотропиномы — это одна из наиболее часто встречаемых опухолей гипофиза в операционном материале. По данным отдела фундаментальной патоморфологии ГНЦ НМИЦ эндокринологии, на долю АКТГ-секретирующих опухолей гипофиза приходится до 39,4% случаев у взрослых и 62,5% у детей. Это позволяет использовать как образцы нефиксированной формалином ткани опухоли, так и из парафиновых блоков в совокупности с исследованием крови для выполнения молекулярно-генетического тестирования.

Использование знаний в отношении генетических аномалий у пациентов с кортикотропиномами начинает входить в клиническую практику. На сегодняшний день наиболее изучены герминальные мутации в опухолях гипофиза, развивающиеся как наследственные заболевания в составе синдромов (различные варианты синдрома множественных эндокринных неоплазий (МЭН), комплекс Карни, синдром МакКьюна-Олбрайта, 3Р ассоциации: феохромоцитома\параганглиома\опухоль гипофиза, DICER1, семейные изолированные опухоли гипофиза). Однако группа этих опухолей достаточно малочисленна и составляет всего 5% от всех новообразований аденогипофиза. Большая часть представлена спорадическими опухолями с наличием соматических мутаций в различных генах и менее изучена по сравнению с аденомами гипофиза, развившихся в составе указанных синдромов.

Рецензируемые исследовательские статьи были взяты из Национального центра биотехнологий «Информационная база данных» (https://pubmed.ncbi.nlm.nih.gov/, за 2018-2022 гг). Основная цель анализа литературы — всесторонний поиск молекулярно-генетических маркеров в отношении гормонально активных и гормонально неактивных кортикотропином из исследований, проведенных в предыдущие годы, поэтому для статей не было установлено ограничений по дате. Ключевые слова для статей по геномике включали: «генетика кортикотропином/аденом гипофиза/нейроэндокринных опухолей гипофиза, семейных кортикотропином/аденом гипофиза/нейроэндокринных опухолей гипофиза», «секвенирование кортикотропином/аденом гипофиза/нейроэндокринных опухолей гипофиза». Для статей по транскриптомике ключевыми словами являлись «транскриптом кортикотропином/аденом гипофиза/нейроэндокринных опухолей гипофиза»», а также «секвенирование РНК кортикотропином/аденом гипофиза/нейроэндокринных опухолей гипофиза». Для классификации различных подтипов нейроэндокринных опухолей гипофиза и выделения среди них вариантов кортикотропином в обзоре использовалась классификация опухолей эндокринных органов Всемирной организации здравоохранения (ВОЗ) от 2022 г.

Цель данной работы — представить результаты комплексного анализа полного экзома и транскриптома гормонально активных и неактивных кортикотропином, а также данные современных технологий, посвященные изучению микроРНКома, ДНК метилома. В то же время в нашей работе мы попытались проследить, каким образом фундаментальные исследования позволяют определить ключевые механизмы патогенеза и биомаркеры кортикотропином, и как возможно использовать эти показатели в клинической практике для скрининга и более ранней диагностики наследственных синдромов и изолированных кортикотропином, дифференциальной диагностики различных форм эндогенного гиперкортицизма, чувствительности к уже существующим и потенциальным видам терапии, определении персонализированного исхода гормонально активных и неактивных кортикотропином.

## Наследственные синдромы с герминальными мутациями

Среди наследственных синдромов для кортикотропином описаны синдромы МЭН1, 2А, 2Б и 4, DICER1, а также синдром семейных изолированных опухолей гипофиза (FIPA), комплекс Карни и синдром Линча.

## Синдром множественных эндокринных неоплазий (МЭН)

Синдром множественных эндокринных неоплазий 1 типа (МЭН1) наследуется по аутосомно-доминантному типу с распространенностью около 1:10 000–1:100 000 населения. Опухоли гипофиза развиваются у 30–40% таких пациентов, тогда как гиперплазия или опухоли околощитовидных желез и нейроэндокринные опухоли желудочно-кишечного тракта возникают гораздо чаще (до 90 и 60% соответственно). Среди опухолей гипофиза в составе синдрома МЭН1 кортикотропиномы развиваются достаточно редко. По данным Vergès B и соавт., их доля составляет около 4%. Наиболее часто диагностируют пролактиномы (40–60%), далее следуют гормонально неактивные опухоли гипофиза (15–40%) и соматотропиномы (5–10%). Клинически такие опухоли характеризуются крупными размерами, чаще поражают молодых пациентов, могут быть множественными или способны секретировать 2 и более гормонов аденогипофиза [1].

У 90% пациентов с МЭН1 синдромом определяется герминальная гетерозиготная мутация в гене MEN1, который расположен на длинном плече 11 хромосомы (11q13) и является геном-супрессором. MEN1 кодирует синтез белка менина, который располагается в ядре клетки и участвует в регуляции клеточной транскрипции, делении и пролиферации клеток, поддержании геномной стабильности. Последние годы появились коммерческие антитела к менину для выполнения иммуногистохимического исследования в ткани опухоли с целью скрининга данной мутации [2].

В литературе описаны единичные случаи кортикотропином (1 у взрослого и 1 у ребенка), развившихся в рамках синдромов МЭН 2А и 2В. Данные синдромы связаны с мутацией онкогена RET. Он кодирует трансмембранный рецептор, связанный с активностью тирозинкиназы, которая действует как протоонкоген [3][4].

Синдром МЭН4 — это редкое генетическое заболевание, которое развивается у 1,5–3% пациентов при отсутствии мутаций в гене MEN1. Патология вызвана мутацией в гене CDKN1B, который расположен в хромосоме 12q13 и кодирует циклин-зависимую киназу р27. Этот фермент регулирует клеточный цикл от G1 до S фазы митоза. В литературе описаны единичные случаи таких пациентов с АКТГ-секретирующими опухолями [5].

DICER1 синдром — это редкое, аутосомно доминантное заболевание, обусловленное герминальной гетерозиготной мутацией в гене DICER1. Синдром включает в себя различные доброкачественные и злокачественные опухоли: плеврально-легочная бластома, опухоли яичников из клеток стромы полового тяжа (сертоликлеточные и лейдигоклеточные), кистозная нефрома, узловая гиперплазия и злокачественные дифференцированные опухоли щитовидной железы, бластома гипофиза, назальная хондромезенхимальная гамартома, медуллоэпителиома реснитчатого тела, почечная саркома, эмбриональная рабдомиосаркома и пинеалобластома. Бластома гипофиза впервые описана в 2008 г. [6], гистологически представлена Ратке-подобными эпителиальными клетками, формирующими розетки или железисто-подобные структуры; среди них имеются АКТГ-позитивные клетки (реже — клетки с экспрессией гормона роста). В составе DICER1 синдрома бластомы гипофиза диагностируют редко (менее 1%). Они характеризуются крупными размерами и тяжелым течением БИК у детей с медианой возраста 8 месяцев (в диапазоне от 7 до 24 месяцев), смертность достигает 40%. Мутация в гене DICER1 является патогномоничной для данного синдрома. Ген расположен на длинном плече 14 хромосомы (14q32.13), кодирует цитоплазматическую эндорибонуклеазу, которая отвечает за созревание и модуляцию экспрессии микроРНК на посттранскрипционном уровне. Недавно в литературе описан DICER1 синдром у молодого взрослого пациента, обусловленный герминальной гетерозиготной мутацией в гене DICER1 [7].

## Синдром семейных изолированных аденом гипофиза (FIPA)

Герминальная мутация в гене AIP, который кодирует белок, взаимодействующий с арилуглеводородным рецептором, может быть причиной возникновения опухолей гипофиза в 15–30% семей с синдромом семейных изолированных аденом гипофиза. Мутация в данном гене чаще описана у пациентов в пораженных семьях с акрогигантизмом, реже — с пролактиномами или в опухолях со смешанной секрецией гормона роста и пролактина. Для этих новообразований характерны крупные размеры, они резистентные к медикаментозной терапии, поражают молодых людей. Крайне редко такие мутации возникают у пациентов с БИК: в литературе описаны единичные случаи у детей и взрослых. В исследование L. Cazabat [8] были включены 443 пациента, у которых определяли наличие мутаций в гене AIP. Среди них выявили 44 пациента с БИК (10%), у троих из которых обнаружили данную мутацию. Эти пациенты были моложе 40 лет (на момент манифестации заболевания возраст составил 13, 21 и 39 лет). Среди пациентов с кортикотропиномами преобладали те, у которых фиксировались микроаденомы (2 микро- и 1 макроаденома), в отличие от больных с другими гистологическими типами макроаденом гипофиза. Были обнаружены нуклеотидные замены по типу миссенс-мутаций (n=2) и сдвигу рамки считывания (n=1) в гене AIP.

Исследованы и описаны несколько механизмов, приводящих к резистентности к аналогам соматостатина у таких пациентов. К ним относят снижение экспрессии ингибирующего G-протеина, фосфодиэстеразы, а также нарушения сигнального пути протеинкиназы А и ZAC1.

Существуют еще 2 наследственных синдрома, в рамках которых отмечают пациентов с кортикотропиномами. Это комплекс Карни и синдром Линча. Комплекс Карни — это наследственный синдром, обусловленный инактивирующими мутациями в гене PRKAR1A, который кодирует альфа-регуляторную субъединицу цАМФ-зависимой протеинкиназы А. В составе комплекса Карни, кроме шванном, миксом и пигментации кожи, диагностируют кортикостеромы. За последние годы также появились упоминания о единичных кортикотропиномах [9].

Синдром Линча развивается в результате герминальных мутаций в так называемых генах репарации ДНК (MSH2, MLH1, MSH6, PMS2 и EPCAM) и ассоциируется с наследственной предрасположенностью к развитию карцином различной локализации. В литературе описаны случаи пациентов с синдромом Линча с наличием инвазивных макрокортикотропином или кортикотрофной карциномой, у которых обнаружили герминальные мутации в генах MLH1 и MSH2 [10][11].

Таким образом, пациенты с кортикотропиномами молодого (менее 30 лет) и детского возраста могут являться кандидатами для молекулярного тестирования с целью идентификации наследственных синдромов. В качестве скрининга возможно проводить иммуногистохимическое исследование на образцах ткани опухоли с антителами к менину (синдром МЭН1), р27 (синдром МЭН4), MSH2, MLH1, MSH6, PMS2 (синдром Линча).

## Соматические мутации в спорадических кортикотропиномах

Наиболее изученными и распространенными среди спорадических АКТГ-секретирующих опухолей гипофиза являются соматические мутации в генах убиквитин-специфической протеазы 8 (USP8), убиквитин-специфической протеазы 48 (USP 48), BRAFV600E, ATRX.

Полное секвенирование экзома выявило, что 20–60% кортикотропином являются носителями соматической мутации в гене USP8. Данная мутация является специфичной для кортикотропином и не обнаружена в других типах опухолей аденогипофиза или АКТГ-эктопических опухолей негипофизарной локализации.

«Горячая точка» гена USP8 расположена в 14 экзоне связывающего домена 14-3-3. В белке USP8 дикого типа в домене 14-3-3 происходят конформационные изменения, которые позволяют USP8 блокировать его собственную каталитическую активность. Потеря связывающего участка 14-3-3 при наличии мутации в гене USP8 повышает их деубиквитиназную активность и обеспечивает доступ к протеазам, которые расщепляют его до С-концевого фрагмента длиной 40 кДА с высокой каталитической способностью. Этот процесс нарушает деградацию EGFR (рецептор эпидермального фактора роста), стимулирует транскрипцию проопиомеланокортина (ПОМК) и синтеза АКТГ в опухоли. Надо отметить, что выраженная стимуляция митотической активности и, как следствие, высокая пролиферативная активность в опухолевых кортикотрофных клетках не обнаружена [12].

Кортикотропиномы, содержащие мутации в гене USP8, чаще возникают у женщин, меньше по размеру и неинвазивные. Уровни секреции АКТГ до операции выше по сравнению с показателями пациентов, у которых кортикотропиномы не обладали мутацией в гене USP8. С другой стороны, случаи с наличием соматических мутаций в гене USP8 сопровождаются более высокими уровнями кортизола после удаления опухоли и как следствие рецидивом или продолженным ростом новообразования. Эти данные подтверждаются наличием указанной мутации у 50% пациентов с продолженным ростом кортикотропином при синдроме Нельсона. В то же время показано, что опухоли без мутаций в гене USP8 обладают более крупными размерами с инвазивным ростом в сфеноидальный синус, ремиссия развивается реже [13].

Что касается детей с наличием БИК, то, по данным Faucz FR [14], кортикотропиномы, несущие соматические мутации в гене USP8, обнаружены у 13 из 45 пациентов (28%). Однако в литературе описан клинический случай кортикотропиномы у 16-летней девушки с наличием гетерозиготной герминальной мутации в гене USP8 [15].

Кортикотропиномы, содержащие мутации в гене USP8, обладают высоким уровнем экспрессии рецепторов соматостатина 5 подтипа и О6-метилгуанин ДНК метилтрансферазы (MGMT), следовательно, чувствительны к терапии пазиреотидом (лиганд к рецепторам соматостатина 5 подтипа) и темозоламидом соответственно.

Мутация в гене USP8 может являться мишенью для лечения с помощью низкомолекулярных ингибиторов, демонстрирующих антипролиферативную и антисекреторную эффективность in vitro. В качестве ингибитора EGFR может использоваться гефитиниб для терапии пациентов с кортикотропиномами, содержащими мутации в гене USP8. Показано прямое ингибирование EGFR в первичных культурах клеток кортикотропином, содержащих мутацию в указанном гене, с последующим ослаблением высвобождения АКТГ [16][17].

Среди кортикотропином с помощью секвенирования следующего поколения (NGS) была выявлена вторая «горячая точка» в гене, кодирующем другую деубиквитиназу, — USP48. Мутация в гене USP48 наблюдаются в 4–23% случаев кортикотропином (без мутаций в гене USP8) и является миссенс-мутацией, включающей M415I/V субституцию. Мутантный ген усиливает транскрипционную активность гена POMC, предшественника АКТГ, за счет митоген-активируемой протеинкиназы (MAPK). Для кортикотропином, несущих мутацию в гене USP48, характерны меньшие размеры и более высокая чувствительность к стимуляции кортикотропин-рилизинг гормона (КРГ-физиологического стимулятора синтеза и секреции АКТГ в кортикотрофных клетках). In vitro на модели клеток кортикотропином (MET415lle) с наличием мутации в гене USP48 определялось умеренное повышение секреции АКТГ. На фоне стимуляции КРГ уровни ПОМК и АКТГ были значительно выше базальных значений, секретируемые клетками кортикотропином с наличием мутации в гене USP48, по сравнению с клетками без мутаций в данном гене (р<0,01). На основании этих данных можно предположить, что мутация в гене USP48 может индуцировать онкогенез кортикотропином за счет высокой чувствительности к стимулу КРГ со стороны гипоталамуса. В экспериментах также было показано значение Gli1 (глиома-ассоциированный онкоген), медиатора сигнального пути Sonic Hedgehog (SHH). Gli1 считается ключевым фактором, который влияет на процесс убиквитинирования и потенцирует синтез АКТГ за счет активации сигнального пути SHH в кортикотропиномах, содержащих мутации в гене USP48. Предполагается подобное действие Gli1/SHH сигнального пути и в кортикотропиномах с наличием мутации в гене USP8 [18][19].

Около 16% случаев кортикотропином содержат мутацию в гене BRAF V600E при отсутствии мутаций в генах USP8 и USP48. Данная мутация усиливает действие протоонкогена с тирозин-киназной активностью, что запускает MAPK-сигнальный путь и активирует транскрипцию гена POMC и синтез АКТГ.

На сегодняшний день существует проблема определения прогноза течения БИК. Анализ совокупности данных об особенностях клинического течения, МРТ (размер опухоли и/или инвазивный рост), гистологические варианты кортикотропином (плотно- и редкогранулированные опухоли, из Круковских клеток), показатели пролиферативной активности (количество митозов и индекс метки Ki-67, экспрессия Р53) могут не соответствовать характеру течения заболевания (ремиссия, персистирующий гиперкортицизм или рецидив заболевания). В исследовании Uzilov V [20] изучали наличие мутации в генах USP8 и TP53 в кортикотропиномах, в том числе и в когорте опухолей с агрессивным течением. В результате было показано, что одновременное содержание указанных мутаций в одной и той же кортикотропиноме исключалось. Это подтверждает независимую работу данных драйверных генов. Многочисленные исследования доказывают наличие мутации в гене ТР 53 во многих карциномах различной локализации. Среди АКТГ-секретирующих новообразований гипофиза обнаружены случаи с инактивирующей соматической драйверной мутацией в гене ТР53. Группа этих опухолей (n=5 из 22) была представлена инвазивными макроаденомами с персистирующим или рецидивирующим течением заболевания. Опухоли были позитивны к P53 (при иммуногистохимическом исследовании) и обладали высоким пролиферативным индексом Ki-67 (более 3%). В 3 случаях диагностировали синдром Нельсона, у 1 пациента выявлена карцинома. На основании этих данных авторы определили взаимосвязь между наличием мутации в гене ТР53 и агрессивным течением БИК, также предположили, что позитивная иммуноэкспрессия Р53 ассоциирована с инвазивным и агрессивным характером роста опухолей. Однако необходимо дальнейшее изучение взаимосвязи между наличием мутации в гене ТР53, позитивной иммуноэкспрессией Р53 в клетках опухоли и агрессивным течением БИК.

Данное исследование также продемонстрировало, что кортикотропиномы, несущие соматические мутации в гене USP8, были более генетически стабильны по сравнению с опухолями без мутаций в этом гене и обладали благоприятным течением БИК. Часть опухолей (22,7%) содержали мутацию в гене TP53 и обладали высоким уровнем хромосомной нестабильности. В этих случаях наблюдалось персистирующее или рецидивирующее течение заболевания.

В ряде работ показана связь с наличием мутации в гене ATRX (alpha thalassemia/mental retardation syndrome X-linked — синдром альфа-таласемии и умственной отсталости X-сцепленного типа), регулирующий ремоделирование хроматина и поддержание структуры и функции теломеры. Инактивация гена ATRX в опухолях приводит к дестабилизации теломеры и стимулирует процесс альтернативного удлинения теломер, что запускает состояние бессмертия опухолевых клеток. Соматические мутации в гене ATRX ассоциируются с астроцитомами у взрослых и такими нейроэндокринными опухолями (НЭО), как НЭО поджелудочной железы, феохромоцитомы/параганглиомы, нейробластомы. Предполагается, что аномалии в структуре гена ATRX являются предикторами агрессивного поведения указанных новообразований [21][22][23][24][25].

В работах Casar-Borota O. и соавт. [26][27], посвященных изучению иммуноэкспрессии белка ATRX при иммуногистохимическом исследовании и мутации гена ATRX (при NGS) в различных опухолях гипофиза, показано выпадение иммуноэкспрессии этого белка именно в опухолях с агрессивным течением (13%) или в карциномах (28%). Причем большинство были представлены кортикотропными опухолями (32%) по сравнению с Pit-1-позитивными новообразованиями (8%) (соматотропиномы, пролактиномы, тиреотропиномы). Отсутствие экспрессии ATRX было подтверждено наличием молекулярных дефектов гена ATRX по типу потери функции (нонсенс-мутации, вставки со смещением рамки считывания, крупными интрагенными делециями практически всего гена — 22-28 экзонов из 36). В результате авторы предложили использовать белок ATRX как иммуногистохимический маркер — предиктор высокоагрессивных или потенциально метастазирующих кортикотропных опухолей гипофиза.

Таким образом, на сегодняшний день актуальным является изучение особенностей клинического течения, гистологических вариантов и прогноза течения заболевания у пациентов с кортикотропиномами, несущими мутации в генах USP8, USP48, BRAF V600E, ТР 53, ATRX. Изучение молекулярно-генетических аномалий приведет к более глубокому пониманию онкогенеза кортикотропином, выявлению дополнительных свойств опухолей, ассоциированных с неблагоприятным течением БИК. Это будет способствовать активному наблюдению за пациентами — носителями мутаций в генах USP8, USP48 и BRAF и своевременному назначению более агрессивной терапии, а белки-продукты мутаций в указанных генах будут являться мишенями для ингибиторов EGFR и BRAF.

## Эпигенетические маркеры кортикотропином

За последнее десятилетие накоплено немало доказательств в отношении нарушения регуляции экспрессии микроРНК (миРНК), участвующей в развитии и прогрессии опухолей гипофиза. МиРНК являются некодирующими РНК молекулами длиной 18-24 нуклеотидов, которые регулируют экспрессию генов на посттранскрипционном уровне и контролируют такие процессы, как дифференцировка, пролиферация и апоптоз в клетке. МиРНК влияют на стабильность и трансляцию матричной РНК (мРНК) при помощи связывания с регуляторными элементами, расположенными в 3`нетранслируемых регионах подконтрольных транскриптов. Эти молекулы существуют как внутри клетки, так и за ее пределами в различных биологических жидкостях (плазма, сыворотка, моча, слюна, семенная жидкость, ликвор и др.). Доступность указанных биологических сред позволяет использовать миРНК, как неинвазивные биомаркеры различных заболеваний, в том числе опухолей. Для каждого вида опухолей гипофиза характерен свой специфический профиль экспрессии миРНК. В ряде исследований показано, что аберрантная экспрессия определенных миРНК ассоциируется с размером опухоли, наличием инвазивного роста и чувствительности к различным видам терапии.

В исследовании Sh. Vetrivel и соавт. [28] изучалась экспрессия миРНК для дифференциальной диагностики БИК и СИК, БИК и АКТГ-эктопического синдрома.

Профили циркулирующих миРНК изучались в образцах сыворотки у пациентов с СИК, БИК и контрольной группы. В результате была идентифицирована миРНК 182-5p в качестве потенциального неинвазивного маркера БИК, которая может использоваться для дифференциальной диагностики различных форм эндогенного гиперкортицизма.

В работе Малыгиной А.А. и соавт. [29] проводился поиск миРНК, специфичных для различных форм АКТГ-зависимого гиперкортицизма (БИК и АКТГ-эктопического синдрома). В результате выявлены возможные кандидаты миРНК (miR-383-3p, miR-1229-3p, miR-1203, miR-639, miR-4290, miR-6717-5p, miR-302c-3p), которые предполагается использовать в качестве инструмента в дополнение к уже известным и широко применяемым методам (МРТ головного мозга и селективный забор крови из нижних каменистых синусов) для дифференциальной диагностики форм АКТГ-зависимого гиперкортицизма [30].

Важно отметить исследование F. Garbicz и соавт. [31], в котором определялась иммуноэкспрессия MCM7 (белок 7 для поддержания мини-хромосом участвует в инициации репликации генома эукариот) и экспрессия кластера миРНК miR-106b~25 (miR-106b-5p, miR-93-5p, miR-93-3p and miR-25-3p) при помощи количественной ПЦР в реальном времени в ткани кортикотропином. МСМ7 является геном-мишенью для кластера миРНК miR-106b~25. В АКТГ-секретирующих опухолях, по данным МРТ, определяли степень инвазивного роста по шкале Knosp. При помощи иммуногистохимического исследования выделяли гистологические варианты кортикотропином (плотногранулированные, редкогранулированные и опухоли из круковских клеток), проводили анализ индекса метки Ki-67 и p53. В результате была выявлена повышенная экспрессия МСМ7 и высокий индекс метки Ki-67 в инвазивных кортикотропиномах и в кортикотропиномах из круковских клеток. Экспрессия miR-93-5p была значительно повышена в инвазивных опухолях по сравнению с неинвазивными. Кроме того, все четыре миРНК из кластера miR-106b~25 продемонстрировали гиперэкспрессию в аденомах из круковских клеток. Примечательно, что MCM7 и miR-106b-5p коррелировали со степенью инвазивного роста, оцененного по шкале Knosp. Комбинация экспрессии MCM7, Ki-67 и кластера miR-106b~25 точно дифференцирует инвазивные опухоли от неинвазивных и обладает значительной дискриминационной способностью прогнозировать послеоперационный рецидив/прогрессирование опухоли.

Таким образом, эпигенетические маркеры обладают специфическими характеристиками, которые позволяют дифференцировать формы эндогенного гиперкортицизма (АКТГ-зависимые и АКТГ-независимые) и являются прогностическими биомаркерами.

## Пангеномная классификация кортикотропином

Ранее в исследованиях использовался моноомиксный подход, основанный на анализируемых клинических или патоморфологических критериях. Последние годы ученые предлагают применять так называемые мультиомиксные методы исследования, которые включают в себя исследование полного экзома и транскриптома. Все эти исследования выполняются при помощи секвенирования следующего поколения (NGS). Т.е. используются комбинации «омиксных» методов с учетом клинической картины, патоморфологических особенностей, характера течения заболеваний (индолентный или агрессивный), чувствительности к различным видам терапии. Мультиомиксный подход в изучении опухолей гипофиза в целом и кортикотропином в частности предполагает детальное понимание онкогенеза. Анализ полного экзома демонстрирует наличие наследственных или соматических драйверных мутаций, хромосомных аномалий. Транскриптом является частью генома, который транскрибируется на РНК с последующей регуляцией генов и синтеза их продуктов (набор белков). МиРНКом представлен гипер- или гипоэкспрессией миРНК. Транскриптом и миРНКом отражает различные свойства опухолей, например происхождение или способность к прогрессии. ДНКметилом демонстрирует уровень гипер- или гипометилирования различных генов, характерных для определенных типов опухолей гипофиза [32].

В исследовании M. Neou [33] проводился совокупный анализ типа секреции опухолей гипофиза, иммунофенотипа, наличие ремиссии или персистенции заболевания, полного экзома и РНК последовательностей, а также эпигенетического профиля и ДНК метилирования. В данной работе на основании комплексного анализа генома опухолей гипофиза было введено такое понятие, как пангеномная классификация. В результате показано, что кортикотропиномы без мутаций в гене USP8 обладали более агрессивным течением с преобладанием инвазивного роста в сфеноидальный синус по сравнению опухолями, содержащими мутацию в гене USP8. Отличия в отношении инвазивного роста в кавернозный синус и уровня индекса метки Ki-67 в этих группах не обнаружены. Инвазивный рост кортикотропиномы без мутации в гене USP8 ассоциировался со способностью этих опухолей к эпителиально-мезенхимальному транзиту (снижению степени дифференцировки). Среди кортикотропином с наличием мутации в гене USP8 выявлен более высокий уровень экспрессии рецепторов соматостатина 5 подтипа по сравнению с опухолями без мутаций в этом гене, тем самым подтверждая потенциальную значимость USP8 статуса в отношении чувствительности к пазиреотиду. При анализе структуры хромосом нарушения в виде потери или увеличения участков были более выражены в гормонально активных кортикотропиномах по сравнению с «немыми» новообразованиями. В данной работе хромосомные нарушения не ассоциировались ни с иммунофенотипом опухоли, ни с агрессивным течением. Профили миРНК и метилирование ДНК коррелировали с секреторными типами опухолей, и кортикотропиномы обладали своим уникальным набором экспрессии этих молекул. Кортикотропиномы продемонстрировали обратную корреляцию между уровнем метилирования и хромосомными аномалиями. Такие характеристики транскриптома, как низкий уровень оксидативного фосфорилирования, воспаления и эпителиально-мезенхимального транзита, значительно отличались между различными типами кортикотропином в зависимости от USP8 статуса. При изучении транскриптома в клинически гормонально неактивных кортикотропиномах были выявлены черты, характерные как для кортикотропином, так и гонадотропином. Эти свойства были подтверждены наличием коэкспрессии иммуногистохимических маркеров, которые выявляются как в гонадотропиномах, так и в кортикотропиномах (АКТГ, TPIT, GATA3). В исследовании Salomon M.P. и соавт. [34] были выявлены особенности в метиломе кортикотропином: определены участки гипометилирования промоторов гена POMC. В то же время проводилось сравнение уровней экспрессии POMC и метилирования промотора гена POMC среди кортикотропином с мутациями и без в гене USP8. В результате разница по указанным показателям в зависимости от USP8 статуса не обнаружена и сделан вывод о том, что ДНК метилирование промотора POMC и последующая его экспрессия и синтез АКТГ происходят независимо от указанного состояния. Совокупность современных молекулярных методов для изучения кортикотропином с учетом использования типов нуклеиновых кислот или РНК молекул, а также существующие дизайны исследований, представлены на рисунке 1.

**Figure:**
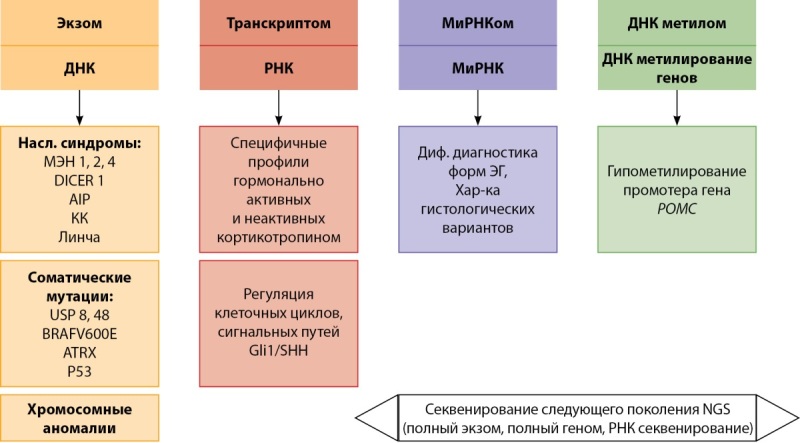
Рисунок 1. Мультиомиксные методы изучения кортикотропином. Примечание: насл. синдромы — наследственные синдромы, КК — комплекс Карни, диф. диагностика — дифференциальная диагностика, ЭГ — эндогенный гиперкортицизм, хар-ка — характеристика. За основу данного рисунка взято изображение из статьи Peculis R, Niedra H, Rovite V. Large scale molecular studies of pituitary neuroendocrine tumors: novel markers, mechanisms and translational perspectives. Cancers. 2021;13,1395. doi: https://doi.org/10.3390/cancers13061395 [32] и переработано автором.

## ЗАКЛЮЧЕНИЕ

Таким образом, многочисленные результаты фундаментальных как моно-, так и мультиомиксных исследований кортикотропином представляют комплексный детальный анализ и позволяют определить новые, ранее не описанные молекулярные варианты опухолей, способствуют более глубокому пониманию биологических механизмов. Это потенциально приведет к усовершенствованию диагностики и лечения пациентов с болезнью Иценко-Кушинга в клинической практике.

## ДОПОЛНИТЕЛЬНАЯ ИНФОРМАЦИЯ

Источники финансирования. Грант Минобрнауки, соглашение №075-15-2022-310 от 20.04.2022.

Конфликт интересов. Автор декларирует отсутствие явных и потенциальных конфликтов интересов, связанных с публикацией настоящей статьи.

Участие авторов. Автор одобрил финальную версию статьи перед публикацией, выразил согласие нести ответственность за все аспекты работы, подразумевающую надлежащее изучение и решение вопросов, связанных с точностью или добросовестностью любой части работы.
